# Author Correction: Corticostriatal activity related to performance during continuous de novo motor learning

**DOI:** 10.1038/s41598-024-56030-4

**Published:** 2024-03-05

**Authors:** Sungbeen Park, Junghyun Kim, Sungshin Kim

**Affiliations:** 1 Department of Artifcial Intelligence, Hanyang University, 222 Wangsimni-Ro Seongdong-Gu, Seoul, 04763 Republic of Korea; 2 Department of Data Science, Hanyang University, 222 Wangsimni-Ro Seongdong-Gu, Seoul, 04763 Republic of Korea; 3 Center for Neuroscience Imaging Research, Institute for Basic Science, 2066 Seobu-Ro, Jangan-Gu, Suwon, 16419 Republic of Korea

Correction to: *Scientific Reports* 10.1038/s41598-024-54176-9, published online 14 February 2024

The original version of this Article contained an error in Affiliation 3, which was incorrectly given as ‘Center for Neuroscience Imaging Research, Institute for Basic Science, 222 Wangsimni-Ro Seongdong-Gu, Suwon, 16419, Republic of Korea’. The correct affiliation is listed below.

Center for Neuroscience Imaging Research, Institute for Basic Science, 2066 Seobu-Ro, Jangan-Gu, Suwon, 16419, Republic of Korea.

The original Article has been corrected.

