# Supplementary material for: Corticostriatal activity related to performance during continuous de novo motor learning
Source: Sci Rep. 2024 Feb 14;14:3731. doi: 10.1038/s41598-024-54176-9 (PMC10867026; doi:10.1038/s41598-024-54176-9)
Supplement: Supplementary file 1 — Supplementary Information. [file 41598_2024_54176_MOESM1_ESM.docx]

**Supplementary Information for**

**Corticostriatal activity related to performance during continuous *de novo* motor learning**

**Short Title:** Corticostriatal activity during motor learning

**Authors:** Sungbeen Park**^1^**, Junghyun Kim^2^, Sungshin Kim^1,2,3^*****

1. Department of Artificial Intelligence, Hanyang University, 04763 Seoul, Republic of Korea
2. Department of Data Science, Hanyang University, 04763 Seoul, Republic of Korea
3. Center for Neuroscience Imaging Research, Institute for Basic Science, Suwon 16419, Republic of Korea

**keywords:** Striatum, motor skill learning, fMRI, visual feedback

*Corresponding Author

222 Wangsimni-ro Seongdong-gu, Seoul, Republic of Korea

Email: sungshinkim@hanyang.ac.kr

Phone: +82-10-4687-5322


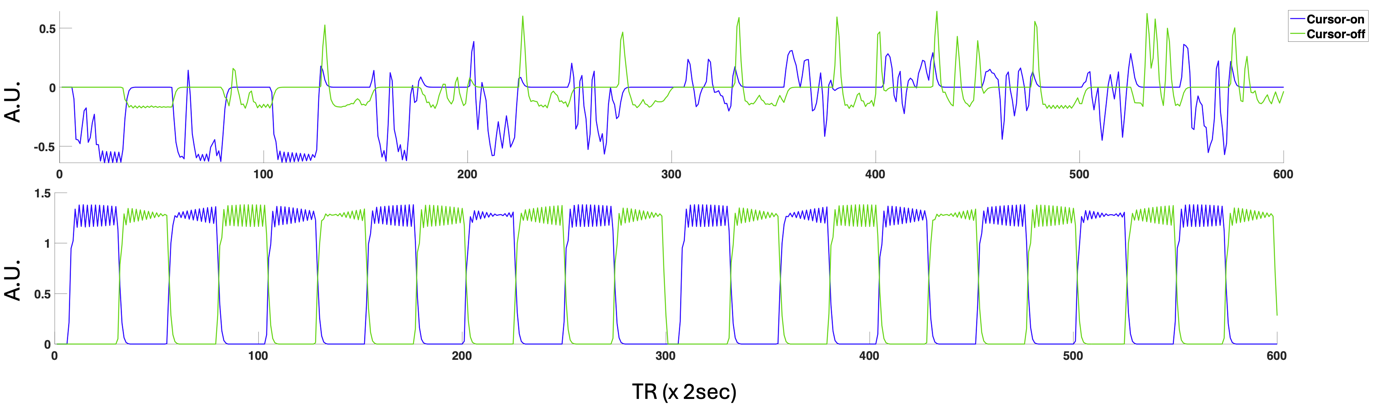


Figure S1. Regressors generated by AFNI program, 3dDeconvolve with an option " stim_times_AM2". Two regressors for each of the feedback conditions, parametric regressor (top) and boxcar regressor (bottom). The parametric regressors capture the fMRI activity modulated by the performance while the boxcar regressors capture the average fMRI activity for separate conditions. Jittering in the regressors was present because the parameter was assessed for each trial (4 sec) during a block of 12 trials without a break between them.


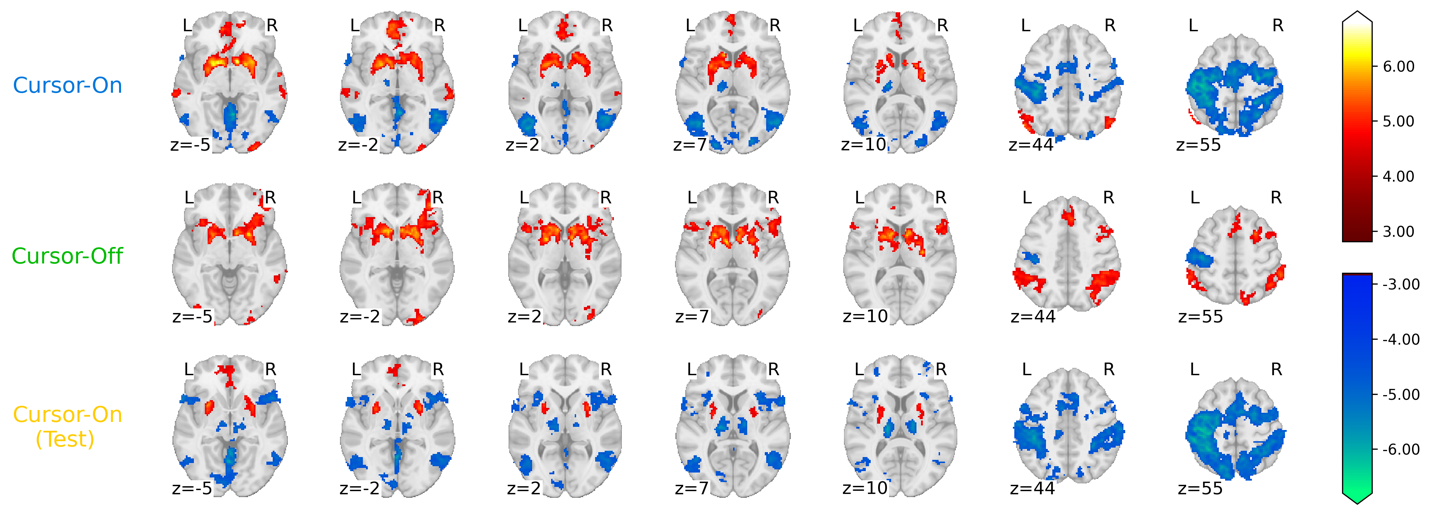


Figure S2. Whole-brain voxelwise GLM analysis for three learning conditions with "cursor-on," "cursor-off," and "cursor-on (test)."


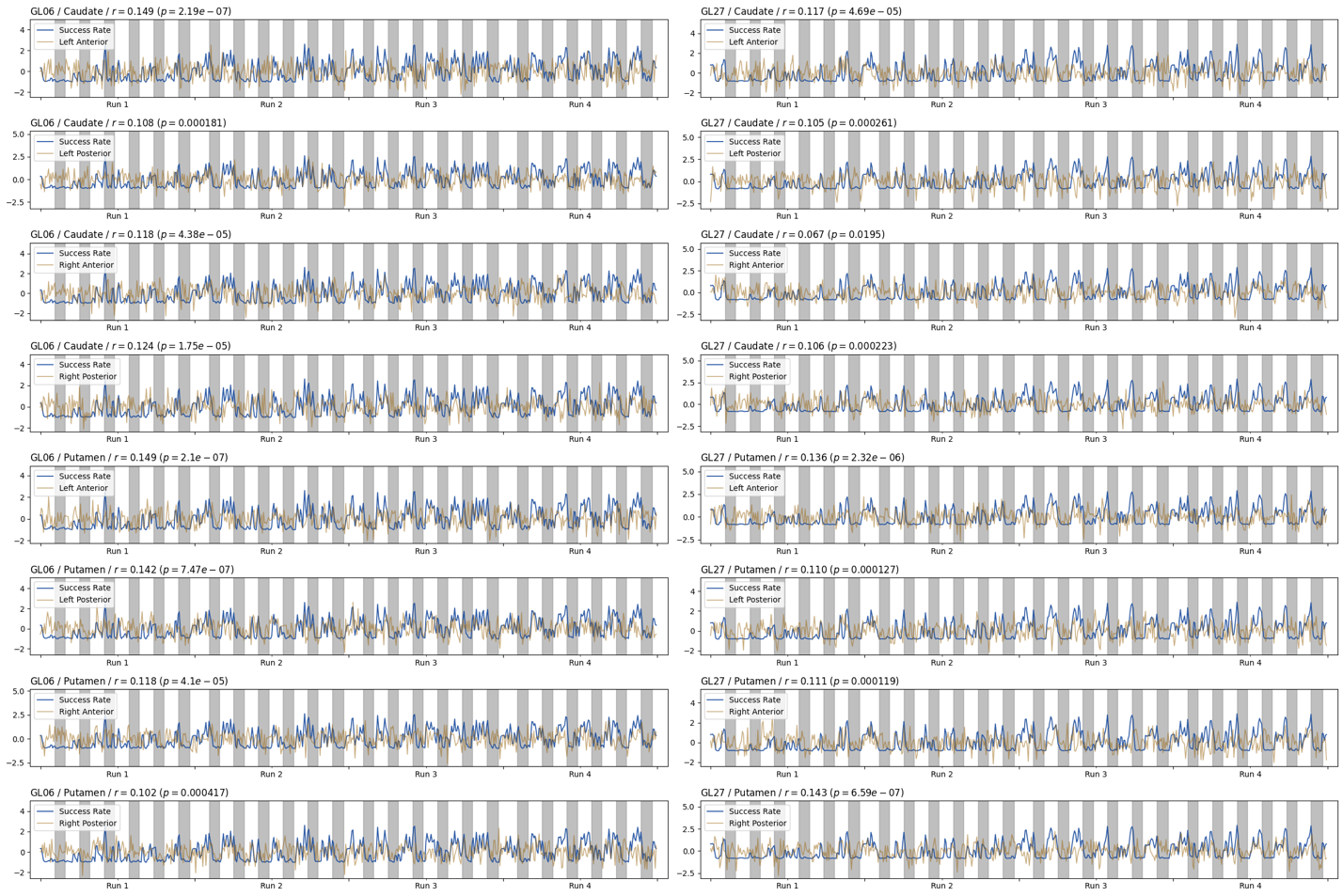


Figure S3. Examples of a parametric regressor which is an HRF-convolved trial-by-trial success rate (blue) and fMRI BOLD signal (gold) extracted from a voxel with a peak response in the caudate nucleus and putamen for two representative participants. Six regressors of non-interest related to the head motion and 4th-order polynomial trends were projected out from the BOLD signals. Then, the BOLD signals were resampled every 4 s (original TR = 2 s) for better comparison with the parametric regressor that modulates the trial-by-trial success rate.


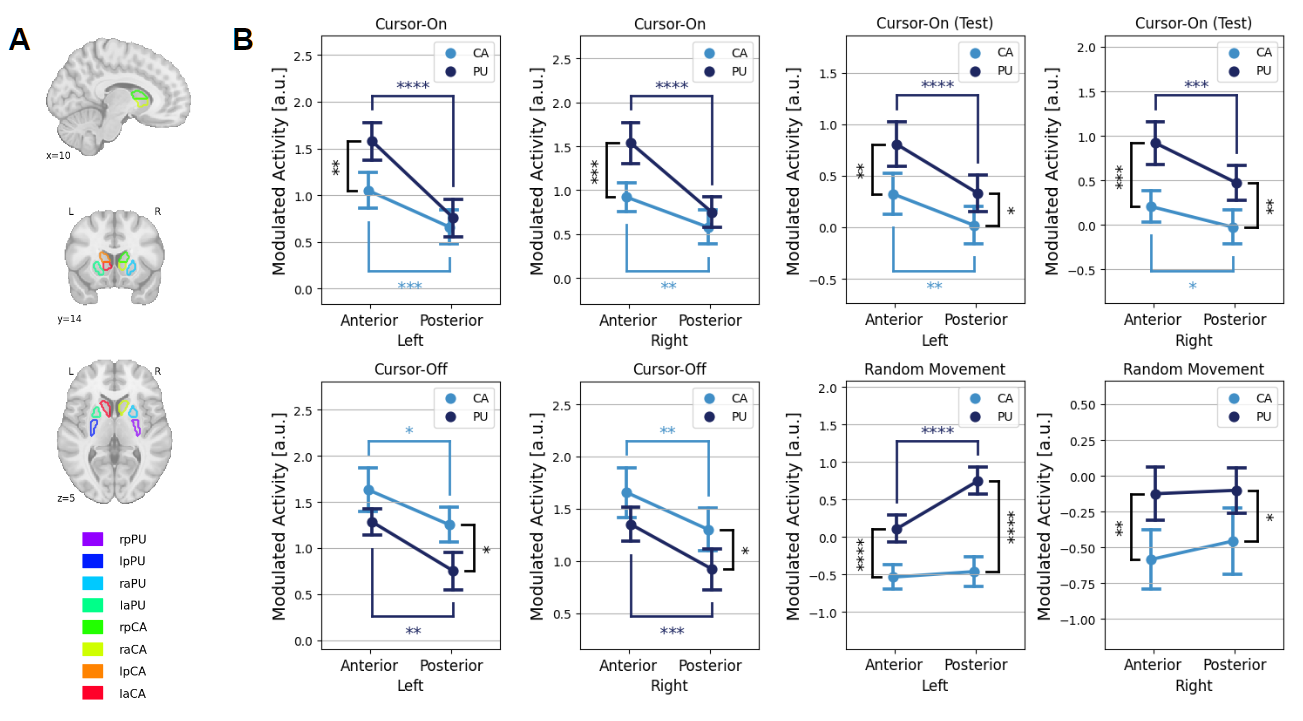


Figure S4. (A) As sub-regions of the striatum, the putamen and caudate were independently split into anterior and posterior portions. Where r stands for right, l for left, a for anterior, and p for posterior. (B) Spatial dissociation of the striatal activity for four different conditions, three conditions for performance-modulated activity "Cursor-On," "Cursor-Off," "Cursor-On (Test)," and one condition for random motor control. Error bars indicate SEM. *p<0.05; **p<0.01; ***p<0.001; ****p<0.0001 (uncorrected p).
